# Towards defining muscular regions of interest from axial magnetic resonance imaging with anatomical cross-reference: part II - cervical spine musculature

**DOI:** 10.1186/s12891-018-2074-y

**Published:** 2018-05-28

**Authors:** James M. Elliott, Jon Cornwall, Ewan Kennedy, Rebecca Abbott, Rebecca J. Crawford

**Affiliations:** 1 Faculty of Health Sciences, The University of Sydney, Northern Sydney Local Health District, St Leonards, Australia 75 East Street Lidcombe NSW, Sydney, 2141 Australia; 2 Department of Physical Therapy and Human Movement Sciences, Feinberg School of Medicine, Northwestern University, Chicago, USA; 3 Honorary Fellow School of Health and Rehabilitation Sciences, The University of Queensland, St. Lucia, Australia; 4 Centre for Early Learning in Medicine, Otago Medical School, University of Otago, Dunedin, New Zealand; 5 School of Physiotherapy, University of Otago, Dunedin, New Zealand; 6 Faculty of Health Sciences, Curtin University, Perth, Australia

**Keywords:** Cervical spine, Paravertebral muscles, Muscle fat infiltration, Magnetic resonance imaging, Region of interest, Manual segmentation

## Abstract

**Background:**

It has been suggested that the quantification of paravertebral muscle composition and morphology (e.g. size/shape/structure) with magnetic resonance imaging (MRI) has diagnostic, prognostic, and therapeutic potential in contributing to overall musculoskeletal health. If this is to be realised, then consensus towards standardised MRI methods for measuring muscular size/shape/structure are crucial to allow the translation of such measurements towards management of, and hopefully improved health for, those with some musculoskeletal conditions. Following on from an original paper detailing methods for measuring muscles traversing the lumbar spine, we propose new methods based on anatomical cross-reference that strive towards standardising MRI-based quantification of anterior and posterior cervical spine muscle composition.

**Methods:**

In this descriptive technical advance paper we expand our methods from the lumbar spine by providing a detailed examination of regional cervical spine muscle morphology, followed by a comprehensive description of the proposed technique defining muscle ROI from axial MRI. Cross-referencing cervical musculature and vertebral anatomy includes an innovative comparison between axial E12 sheet-plastinates derived from cadaveric material to a series of axial MRIs detailing commonly used sequences. These images are shown at different cervical levels to illustrate differences in regional morphology. The method for defining ROI for both anterior (scalenes group, sternocleidomastoid, longus colli, longus capitis) and posterior (multifidus, semispinalis cervicis, semispinalis capitis, splenius capitis) cervical muscles is then described and discussed in relation to existing literature.

**Results:**

A series of steps towards standardising the quantification of cervical spine muscle quality are described, with concentration on the measurement of muscle volume and fatty infiltration (MFI). We offer recommendations for imaging parameters that should additionally inform a priori decisions when planning investigations of cervical muscle tissues with MRI.

**Conclusions:**

The proposed method provides an option rather than a final position for quantifying cervical spine muscle composition and morphology using MRI. We intend to stimulate discussion towards establishing measurement consensus whereby data-pooling and meaningful comparisons between imaging studies (primarily MRI) investigating cervical muscle quality becomes available and the norm.

## Background

Magnetic resonance imaging (MRI) has been widely and variably utilised to qualify and quantify musculoskeletal pathology involving a number of soft-tissues in both traumatic [1–6] and non-traumatic [7, 8] neck disorders. Such methods have provided convergent [9, 10] and divergent [11–15] evidence around insight into tissue composition, disease characterisation, response to injury, and changes in somatic and nervous structures potentially due to biological, psychological, and socioenvironmental stresses. Advances in MRI technology have raised the number of investigations quantifying skeletal muscle composition (MFI) and structure (volume, cross-sectional area (CSA)), but not without equivocal results [9]. This variability in findings is likely the result of methodological differences across research groups, including variables such as study design, participant demographics (trauma vs. non-trauma; sex, sociocultural, age range), measurement techniques, and MR parameters used by investigators.

In order to better understand the influence of muscle composition and structure on cervical spine health, it is imperative that clinical research communities explore and establish common methodologies in order to facilitate standardisation and accurate comparison of data between studies. Doing so should ultimately result in an improved understanding of the aetiological features of muscle composition and facilitate an improved prognostic, diagnostic, and theranostic landscape.

While data for age-related, degenerative changes of tissues (e.g. vertebrae, joints, discs, muscles) of the lumbar and cervical spine have been published [16–27], studies assessing age-related alterations in paravertebral muscle morphology [19, 28, 29] remain unique to the healthy lumbar spine. Such normative data, to our knowledge, does not exist for the cervical spine. While cross-sectional and longitudinal studies indicate a positive relationship between MFI and traumatic neck pain (e.g. whiplash associated disorders) [1–3, 5, 6, 30], inconsistent associations are also reported [11–14]. Such inconsistencies have not improved our mechanistic understanding of changes in muscle composition in both traumatic and non-traumatic neck pain. Future works must collectively control for what might be considered normative age-related changes [19, 29, 31], degenerative features of the vertebrae or discs [13, 26, 31–34], and spinal curvature [35–37].

### A way forward through standardisation of methodology

In order to facilitate widespread adoption of agreed and time-efficient techniques for measuring cervical spine muscle quality, a standardised, reliable, and replicable method is urgently required. While there is a general trend toward optimising automated methodologies that quantify muscle composition based on differential tissue signal intensities of paravertebral muscle, even the latest, time-efficient tools require a degree of manual input for defining regions of interest (ROI) [3, 5, 6, 38–41]. A standardised ROI method is arguably most important for these studies where it has been speculated that difficulties identifying morphology of both the cervical and lumbar musculature results in poorer repeatability [6, 38]. With continued improvements in both the uptake of, and imaging quality from, MRI technology, an agreed analysis plan utilising a common research measurement method for the identification of ROIs could result in meaningful comparisons with a target towards knowledge transfer and clinical translation of muscle imaging. Following on from the recent manuscript detailing a method for determining ROI in the lumbar spine [42], the purpose of this proposed method is to provide a standardised MRI procedure for measuring cervical spine muscle composition. The method also serves to initiate and continue discussion on the analysis of skeletal muscle composition amongst and between the global clinical and scientific communities.

## Method

### Challenges for producing a region of interest of cervical muscles using MRI

A number of conventional MRI applications (T1, T2, proton-density, Gradient Echo) are available and have been used to qualitatively and quantitatively measure the water and fat species of healthy and diseased soft-aqueous skeletal muscle tissue [1, 3, 41, 43–49]. Technological advancements have also produced alternatives that can be used to image muscle, such as dual acquisition methods, where frequency is selectively excited to produce a water image [50] and a standard image of fat and water. This, however, produces a challenge when measuring a redundant and anatomically complex set of multi-layered (and small) muscles in the cervical spine. The challenge is further compounded by the advent of higher field scanners (e.g. 3–7 Tesla), where a uniform frequency difference between fat and water content may be difficult, but certainly not impossible, to achieve.

Despite recent technological advances that have permitted further insight into muscle composition, the mechanisms underlying muscle degeneration and their influence on outcomes in neck disorders remain elusive. In addition, the vast majority of symptomatic and asymptomatic population-based studies examining pathoanatomical features (e.g. the intervertebral disc, ligaments, and the skeletal vertebral column) of the cervical spine have used a variety of conventional MRI sequences [1, 2, 12, 13, 26, 30, 34, 51–55]. Despite the large repository of available works, the data derived from these imaging investigations have not revealed a consistent structural lesion(s), or response to said lesion(s), that have clarified the clinical presentation of traumatic or non-traumatic neck disorders. This has, in our opinion, created a clinical (and research) impasse that we believe is due partly to the heterogeneous methods across a number of high quality studies investigating the usefulness of imaging for understanding spinal pathology*.* Ultimately, the clinical value of imaging findings of spinal pathology and/or muscle degeneration will be realised if such findings predict important outcomes or help to identify patients likely to respond to specific interventions (e.g. spinal phenotypes).

Research efforts that focus on the consistent assessment of spinal muscle quality with MRI may improve our collective biological understanding of traumatic and non-traumatic neck disorders and why some, but not others, recover spontaneously. Accordingly, a robust and easily-replicated platform for acquiring, assessing, measuring, analysing, and interpreting imaging data on muscle composition and morphology is needed. Currently a wide variety of methods are used to describe the composition and morphology of cervical spine muscles (see Table 1 for a non-exhaustive summary). This represents a key challenge for both producing consistent regions of interest of cervical spine muscles and allowing comparison between research studies.

**Table 1:** A non-systematic summary of methods across investigations describing cervical spine muscle analysis using magnetic resonance imaging (MRI)

Citation	Reliability	MRI Sequence	Slice Selection	Muscles of Interest	ROI Selection	Fat Detection	Measure
Elliott et al., 2006 [1] Elliott et al., 2008 [7] Elliott et al., 2009 [78] Elliott et al., 2011 [2]	Inter-rater (0.94) Intra-rater (0.94)	T1	Axial images aligned parallel to C2–3 disc; Measured at single slice per level C3-C7; most cephalad slice of each vertebral body selected	MF SSCerv SSCap SpCap UT	Manual	Quantitative Pixel Intensity	Fat Infiltration %MFI = (muscle signal)/(fat signal)*100
Fernandez De Las Penas et al., 2007 [79]	Inter-rater (0.80–0.98)	T1	Axial images aligned parallel to C2–3 disc; measured at single slice per level; most cephalad slice of each vertebral body selected	SSCap SpCap	Manual	N/A	CSA
Elliott et al., 2007 [47]	Intra-rater (0.84–0.99) Inter-rater (0.89–0.96)	T1	Axial images aligned parallel to C2–3 disc; measured at single slice per level C3-C7; most cephalad slice of each vertebral body selected	MF SSCerv SSCap SpCap UT	Manual	N/A	CSA
Okada et al., 2011 [80] Matsumoto et al., 2012 [12]	Intra- rater (0.90) Inter-rater (0.844)	T2	Measurements from a single axial slice aligned parallel to each IVD C3–4, C4–5, and C5–6	MF SSCerv SSCap SpCap	Manual	N/A	CSA
Ulbrich et al., 2012 [14]	Inter-rater (0.79–0.98)	STIR	Axial images aligned perpendicular to the vertebral body in the middle of a 20-slice slab. 2 or 3 overlapping slabs used; measurements from single slice per vertebral level C2, C4, and C5	Deep Extensors All Extensors SCM	Manual	N/A	CSA
Elliott et al., 2013 [41]	Inter-rater for fat-water sequence (0.83–0.99)	T1 vs. Dixon	Axial images aligned perpendicular to the spinal cord at the C2-C3 IVD; measurement from single slice per vertebral level C3-C7	MF	Manual	Quantitative Pixel Intensity vs. Fat (F)-Water (W)	Fat Infiltration % MFI = (fat signal)/(fat signal + water signal) *100
Elliott et al., 2014 [8]	From Elliott et al., 2007 [47] for CSA Intra- rater (0.84–0.99) Inter-rater (0.89–0.96)	T1	Axial images aligned parallel to C2–3 IVD; measurements from single slice crossing IVDs C2-C3 and C5-C6	MF SSCerv SSCap SpCap LCap/LCol SCM	Manual	Quantitative Pixel Intensity	Fat Infiltration %MFI = (fat signal)/(fat signal + water signal)*100 CSA
Elliott et al., 2015 [3]	Not reported	Dixon	Measurements from single slice per vertebral level C3-C7; alignment and slice selection not reported	MF	Manual	Fat-Water	Fat Infiltration %MFI = (fat signal)/(fat signal + water signal)*100
Abbott et al., 2015 [6]	Intra-rater (0.98) Inter-rater (0.93)	Dixon	Measurements averaged over 5 slices for each vertebral level C3-C7; Slab alignment not reported.	MF + SSCerv (combined)	Manual with automatic quartile measure	Fat-Water	Fat Infiltration %MFI = (fat signal)/(fat signal + water signal)*100
Karlsson et al., 2016 [5]	For muscle fat Intra-rater (0.81–0.93) Inter-rater (0.82–0.97)	Dixon	Axial images aligned parallel to vertebral segments; measurements averaged over 5 slices for each vertebral level C4-C7	MF	Manual	Fat-Water	Fat Infiltration %MFI = (fat signal)/(fat signal + water signal)*100 CSA
Au et al., 2016 [57]	Not reported	T1	Axial images aligned parallel to C2–3 intervertebral disc; 3D reconstruction	IC, IS, LS, LoCap, LoC, LCap, LCol, MF, LoCap, LoCerv, SSCap, SSCerv, SCM, UT	Manual	N/A	N/A
Fortin et al., 2017 [27] Fortin et al., 2018 [81]	From [81]:Intra-rater (0.83–0.99) Inter-rater (0.38–0.98)	T2	3D multiplanar reconstruction to align images perpendicular to muscle mass; measurements from a single slice per IVD C2–3 through C6–7	MF SSCerv SSCap SpCap	Manual ROI with semi-automatic muscle/fat thresholding technique	Gray-scale threshold technique to calculate CSA of fat within total muscle CSA; gray-scale range determined for each slice individually	Total CSA Functional CSA (FCSA) (= fat free area) Fatty Infiltration = FCSA/Total CSA
Inoue et al., 2012 [82]	Intra-rater (0.85)	T1 T2-fat suppression	Measured from single slice per level; most caudal slice of C3 and most cephalad slice of each vertebral body C4-C7 selected; slab alignment not reported	MF	Manual	Lean muscle CSA: ROI drawn on T1-W images not including fat Total muscle CSA: ROI drawn on fat suppression T2-W image including fat Fat CSA = Total CSA – Lean Muscle CSA	Fatty Infiltration = (Fat CSA)/(Total muscle CSA)
Mitsutake et al., 2016 [83]	Intra-rater 0.85–0.94 Inter-rater (0.84–0.89)	T1	Measured from single, most cephalad slice at level of injury (C4, C5, or C6)	MF	Manual	Quantitative Pixel Intensity	MFI index = Muscle signal/Fat signal
Abbott et al., 2017 [67]	Intra-rater (0.77–0.88) Inter-rater (0.67–0.82)	Dixon	Axial images aligned parallel to each IVD; measured from 5 slices across each vertebral level C4-C7	MF	Manual	Qualitative grading (0, 1, 2) for each 8 regions within visualized ROI on fat image	Fat Infiltration MFI Score: 0 = no or marginal fat 1 = light fat 2 = distinct fat Sum of scores Total # of 2’s
Choi et al., 2016 [84]	Inter-rater (0.82)	T1	Axial images aligned parallel to the inferior end plate of each vertebral body from C4–5 to C7-T1; measured from single slice per vertebral level	Flexor Group: LCap + LCol Extensor Group: MF + SSCerv	Manual	N/A	CSA Normalized Extensor CSA = (Extensor Muscle CSA)/(Vertebral body CSA) *100
Cagnie et al., 2009 [85]	Inter-rater (0.91)	T1	Measured from a single slice aligned parallel IVD at C4-C5	LCap LCol UT LS SpCap SSCerv MF	Manual	Quantitative Pixel Intensity	Muscle/Fat Index = Muscle signal/Fat signal
Uthaikup et al., 2017 [86]	Intra-rater (0.75–0.96) Inter-rater (0.84–0.99)	T1	Axial images aligned parallel to the C2–3 IVD; measured from a single slice at each vertebral level C2-C3	MF SSCap SpCap LCap LCol SCM	Manual	Quantitative Pixel Intensity	Fat Infiltration MFI = Muscle signal/Fat signal

### Anatomically defining the muscles of interest

The muscles spanning the mid-to-lower cervical spine that are typically examined include: multifidus, semispinalis cervicis, semispinalis capitis, splenius capitis, scalenes, levator scapulae, sternocleidomastoid, and longus capitis and longus colli. We do not describe muscles of the suboccipital region (rectus capitis posterior major and minor, and the superior and inferior obliquus muscles [56]) as it is not possible to accurately measure a clinically useful ROI of the suboccipital muscles from the typically employed transverse images used for assessing cervical musculature. This is because no suboccipital muscle has a long axis close to perpendicular to the transverse plane, thus making measurement of useful cross-sectional ROI impractical. Further, fan shaped muscles such as both rectus capitii muscles require special consideration in order to validate useful measures, given a single cross-sectional measurement along the length of either muscle would pose difficulty for determining whole muscle volume. Future work should include developing imaging protocols for the suboccipital muscles as they require more nuanced imaging methods and measures with careful consideration around the highest resolution possible within a reasonable scan time.

The anatomical study we use and recommend for reference are those detailed in Au et al. [57]. They have provided a comprehensive series of labelled axial MR images from one individual to serve as a reference atlas of the cervical spine musculature to guide clinicians and researchers in the accurate identification of these muscles on MR imaging. We have further reinforced by cross-referencing with the E-12 plastinates that have previously been used to assist morphological studies [42, 58].

### Anterior muscles

#### Sternocleidomastoid (SCM)

**Fig. 1:**
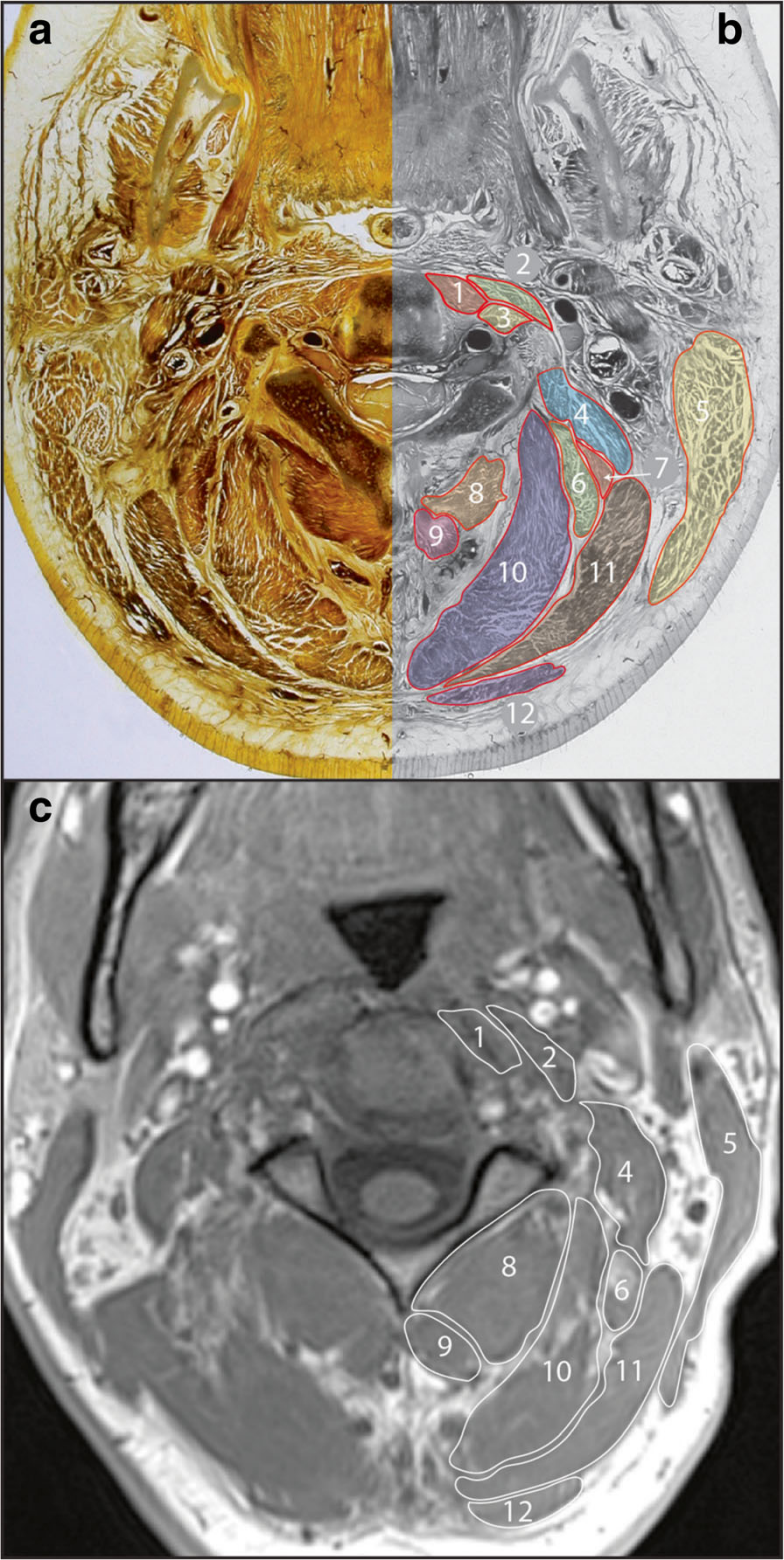
Axial E12 plastinated section (**a**) with schematic illustration (**b**) and in-phase magnetic resonance image (**c**) at approximately C2/3 identifying musculature at this vertebral level. 1. Longus colli; 2. Longus capitis; 3. Intertransversarii; 4. Levator scapulae; 5. Sternocleidomastoid; 6. Longissimus capitis; 7. Splenius cervicis; 8. Inferior obliquus; 9. Rectus capitis posterior major; 10. Semispinalis capitis; 11. Splenius capitis; 12. Trapezius

The SCM arises from the manubrium and medial clavicle inferiorly, and angles laterally and posteriorly towards its superior attachments at the mastoid process and superior nuchal line. This superficial muscle is readily identifiable in cross-section. While the SCM has four portions, [59] as they cross and blend, they are not separable in cross-section along their length on MRI. The muscle has an oval appearance inferiorly, and superiorly forms a distinctive ‘comma’ shape (Fig. 1).

#### Scalenus muscles

Scalenus anterior arises from the scalene tubercle on the first rib as a thin tendon antero-lateral to the lung and pleural cavities, and extends superiorly to attach to the anterior tubercles of the C4–6 (and frequently C3) transverse processes. At the level of the first rib the subclavian vein passes anterior to scalenus anterior, while the subclavian artery passes between scalenus anterior and medius, visibly separating these two muscles. At this level scalenus anterior appears rounded in cross-section. Scalenus medius arises from the first rib posterior to the groove for the subclavian artery and extends superiorly to attach to the transverse processes of C1–7.

#### Longus capitis

**Fig. 2:**
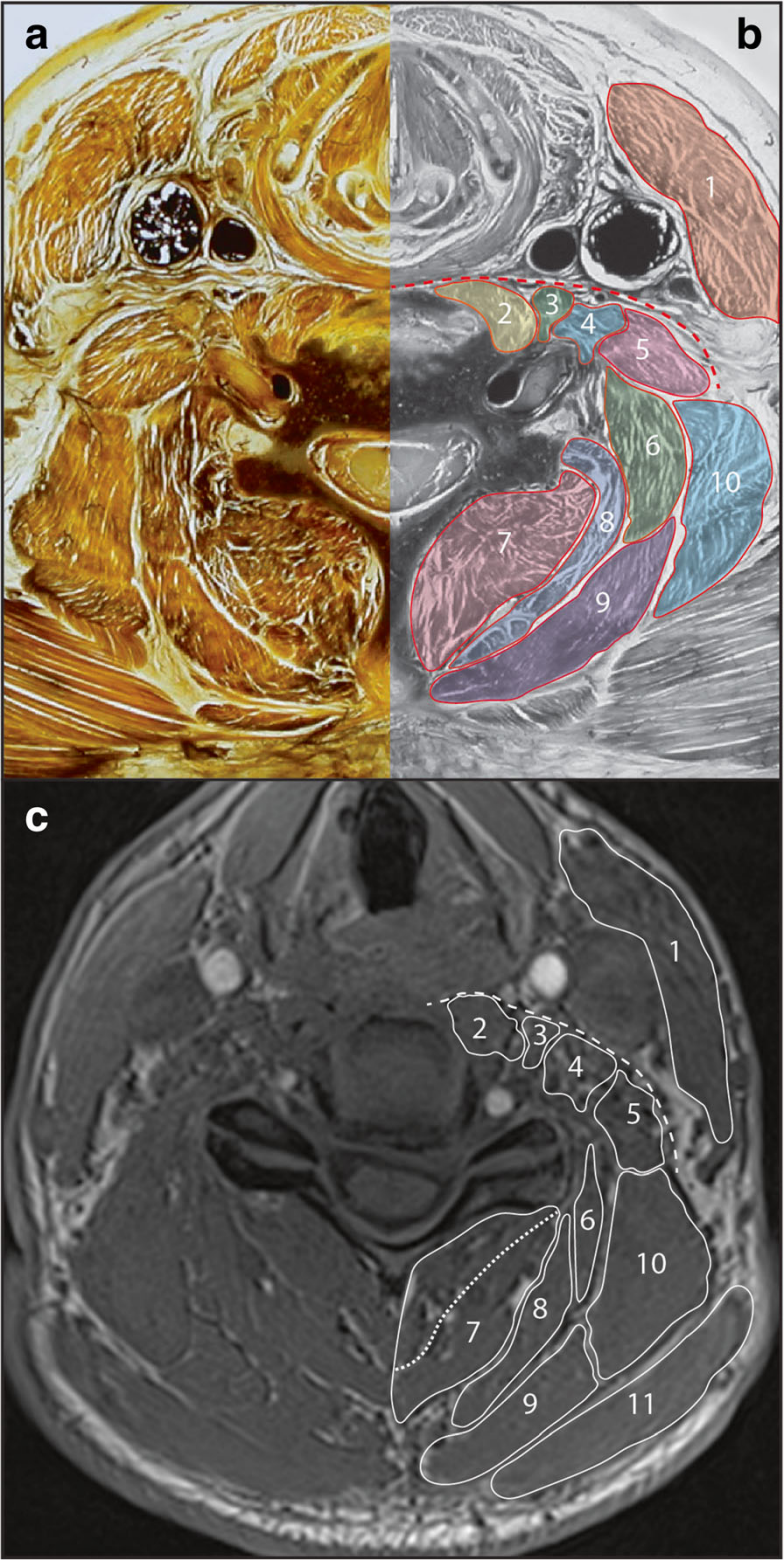
Axial E12 plastinated section (**a**) with schematic illustration (**b**) and in-phase magnetic resonance image (c) at approximately C5/6 identifying musculature at this vertebral level. Dashed red (**b**) and white (**c**) line indicates an anatomical plane which can be used as a reference point for identifying some anterior muscles. Dashed white line in (**c**) indicates likely border between multifidus and semispinalis cervicis. 1. Sternocleidomastoid; 2. Longus colli; 3. Longus capitis; 4. Scalenus anterior; 5. Scalenus medius; 6. Splenius cervicis; 7. Multifidus / semispinalis cervicis; 8. Semispinalis capitis; 9. Splenius capitis; 10. Levator scapulae; 11. Trapezius

This muscle is largest at C1, and has a flattened appearance immediately anterior to the lateral masses on each side of the midline. Inferiorly, it remains anterior to the anterior tubercles of the transverse processes, which allows it to be differentiated from longus colli and the scalenus muscles, particularly scalenus anterior (Fig. 2) [60].

#### Longus colli

Longus colli is recognised by its location in the groove formed between the vertebral bodies and transverse processes of the vertebrae, extending between C1 and T2/3. While longus colli is described as having superior, vertical, and inferior oblique portions, these are based on attachment sites and are not discernible in cross-section [61]. The muscle first becomes visible at C2, emerging medial to longus capitis and initially with a more rounded appearance. Inferior to C7 the muscle thins and moves towards the midline, before attaching to the anterolateral vertebral bodies. Fascial borders between longus colli and the intertransversarii muscles may not be readily apparent between any of the cervical levels on MRI. This should not, however, present difficulties as long as the bony transverse processes are well visualised. Longus colli remains immediately anterior and medial to the bony transverse processes. The intertransversarii muscles are only seen in slices between transverse processes (Fig. 1).

### Posterior muscles

#### Multifidus and rotatores

**Fig. 3:**
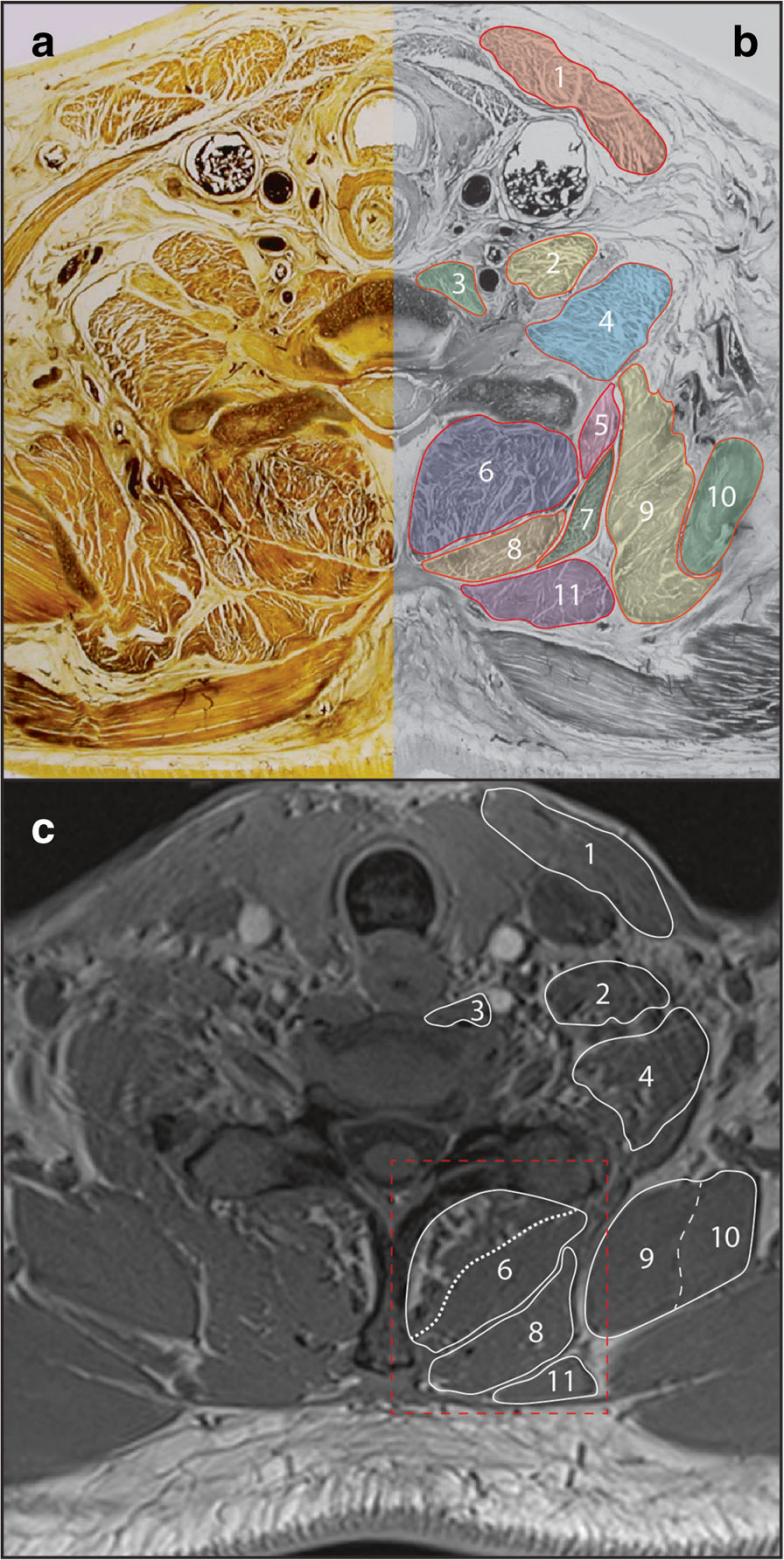
Axial E12 plastinated section (**a**) with schematic illustration (**b**) and in-phase magnetic resonance image (**c**) at approximately C7/T1 identifying musculature at this vertebral level. Red box indicates boundary for Fig. 4. 1. Sternocleidomastoid; 2. Scalenus anterior; 3. Longus colli; 4. Scalenus medius; 5. Iliocostalis cervicis; 6. Multifidus / semispinalis cervicis; 7. Serratus posterior superior; 8. Splenius capitis / cervicis; 9. Levator scapulae; 10. Serratus anterior; 11. Rhomboid minor

Deep against the vertebra, these architecturally complex muscles fill the space between the spinous and transverse processes. Multifidus is present along the length of the spine below C2, forming the deepest layer (Figs. 2, 3). Rotatores can be considered together with multifidus in this deep muscle layer, as these muscles are small and do not form a distinct layer able to be identified in cross-section. Together with semispinalis cervicis, multifidus sits in the paravertebral gutter between the spinous and transverse processes. Because of the intimate relationship between these two muscles [62], it can be difficult to identify them as separate entities on both E12s and MRI.

#### Semispinalis cervicis

Semispinalis cervicis extends between the spinous processes of C2–5 and the transverse processes of T1-T5 [63] (Figs. 1, 2, 3). It overlies multifidus along with other cervical-attaching erector spinae (longissimus cervicis, iliocostalis cervicis). The semispinalis cervicis and erector spinae muscles are difficult, if not impossible, to adequately distinguish in cross-section. The close approximation, similar alignment and attachments of multifidus, semispinalis and erector spinae fascicles are such that a distinct layer will not always be clear on MRI. In this situation it is reasonable to consider these muscles together as a single group (seen [64] and [6]).

#### Semispinalis capitis

Semispinalis capitis is a major muscle of the cervical spine, overlying semispinalis cervicis and forming a large and distinct muscle layer. While semispinalis capitis spans between the occiput and T6–7 [63], in cross-section this layer is most apparent between the occiput and C6/7. Below this level this muscle layer becomes less distinct as semispinalis thins and becomes tendinous towards the thoracic transverse processes (Figs. 1, 2).

#### Erector spinae

Longissimus cervicis extends between the thoracic transverse processes of T1–4 and the C2–6 transverse processes, while iliocostalis cervicis passes between the angles of ribs 3–4 and the transverse processes of C4–6 [63]. As noted, erector spinae muscles attaching to the cervical spine are unlikely to be differentiated from semispinalis cervicis. Longissimus capitis is more distinct, extending between the mastoid process and the transverse processes of approximately C4-T4 (Fig. 1) [63].

#### Splenius capitis and cervicis

Splenius capitis and cervicis form a single layer and overlie semispinalis capitis. Splenius capitis spans between the C7-T4 spinous processes and the mastoid process / occiput, while splenius cervicis spans between the T3–6 spinous processes and the transverse processes of C1–3 [63]. In cross-section, splenius capitis forms a distinct layer between trapezius and semispinalis capitis. Splenius cervicis can be identified between C2–6 on the antero-lateral edge of this layer (Figs. 1, 2, 3), as it diverges from splenius capitis towards its cervical attachments. Below the level of approximately C5 splenius cervicis is unlikely to be visibly separate from splenius capitis in cross-section.

#### Levator scapulae

Levator scapulae have a presence throughout the cervical spine, and its presence is worth noting as one of the larger and more distinctive muscles in the region. It passes from the upper aspect of the medial scapula to the transverse processes of C1–4 [63]. In cross-section levator scapulae is well-defined at lower levels, sitting anterior to trapezius and lateral to splenius (Figs. 2, 3). Superiorly, levator scapulae extends towards the transverse processes of C1–4 in close relation to the scalenus and longus capitis muscles (Fig. 1).

## Results

Our method provides anatomical reference between MRI imaging and E12 plastinates (derived from cadavers) to advance ROI identification and definition to improve standardised measurement of musculature traversing the cervical spine. The E12 plastinates provide a unique opportunity to detail specific tissues that may be MR invisible, [65] leading to natural disagreement across studies where fat-water separation is a target. To follow, we also include suggestions on operational characteristics for acquiring MR images.

### Defining the regions of interest from MRI

Similar to that reported for the lumbar spine, [42] a standard scout image from the sagittal localiser or conventional T2-weighted scan can be used to cross-reference and discern cervical level from axial MR. Users will also find it useful to scroll between the adjacent axial slices to accurately landmark anatomical structures when producing ROIs. The method is applicable to studies examining paravertebral ROIs for single (cross-sectional) or multiple (volumetric) slices. Previous work from the lumbar spine suggests a randomised approach for starting with either the left or right side, and/or separate muscles can influence repeatability when creating ROIs [38, 66]. The same randomised approach is suggested for the cervical spine.

Definitions for ROI measures from MRI for the multifidus, semispinalis cervicis, semispinalis capitis, longissimus capitis, splenius capitis and cervicis, levator scapulae, longus colli, longus capitis, scalenus and sternocleidomastoid are included, describing the anatomical borders (cross referenced to Figs. 1, 2, 3). ROI definitions are detailed with particular reference to cervical levels C2/3, C5/6, and C7/T1. Technical notes are also provided where identifying the guided ROI on MRI may be difficult.

### Anterior muscles

It is worth noting that an anatomical plane that passes laterally and posteriorly in an arc from the anterior aspect of the vertebral body presents a reliable reference point for identifying the anterior aspect of all anterior muscles apart from the sternocleidomastoid (Fig. 2).

#### Sternocleidomastoid

This definition can be applied along the full extent of sternocleidomastoid, from the occiput to approximately T2/3. The anatomical boundaries of sternocleidomastoid are straight forward, and tracing should present few challenges. Some care is needed to trace along the full occipital extent at higher levels (Fig. 1).

#### Scalenus muscles

This definition is best applied at the C6-T2 levels. The scalenus muscles are best identified at their inferior extent arising from the first rib. Superiorly scalenus anterior and scalenus medius converge, and may be difficult to differentiate above the level of C6 on MRI. Differentiation is aided by the angle each muscle approaches the cervical transverse processes, as each muscle follows a straight course. Sequentially from anterior to posterior: longus capitis is seen anterior to the anterior tubercles, scalenus anterior angles to attach to the anterior tubercles slightly more laterally, scalenus medius angles between the anterior and posterior tubercles, scalenus posterior (if present) angles towards the posterior tubercles, and (above C4) levator scapulae also angles to attach to the posterior tubercles (Fig. 2).

#### Longus capitis

This definition is best applied at the C1–5 levels. Longus capitis is largest and most distinct superiorly, just below where it attaches to the basi-occiput. As such, the muscle is best tracked inferiorly from this point. At its superior extent longus capitis has a rounded appearance, which flattens and thins out over the lateral masses of C1. By the level of C2/3 longus capitis is a relatively thin slip immediately anterior to the anterior tubercles of transverse processes C3–6. As for the scalenus muscles, identification is aided by identifying the transverse processes (in particular the anterior tubercles) and remaining posterior to the prevertebral fascia (Fig. 2).

#### Longus colli

This definition is best applied at C2-T1 levels. As noted anatomically, longus colli sits in the groove between the vertebral bodies and transverse processes of the vertebrae. Thus, these bony landmarks must be well visualised to accurately identify the muscle. As described for multifidus, the ROI should closely follow the bony vertebrae to include fat adjacent to the bone. If the anterior aspect of the transverse processes are not visible, or slices above and below are not reviewed to clarify the position of bony landmarks, a ROI for longus colli may be inaccurate.

### Posterior muscles

#### Multifidus and semispinalis Cervicis

**Fig. 4:**
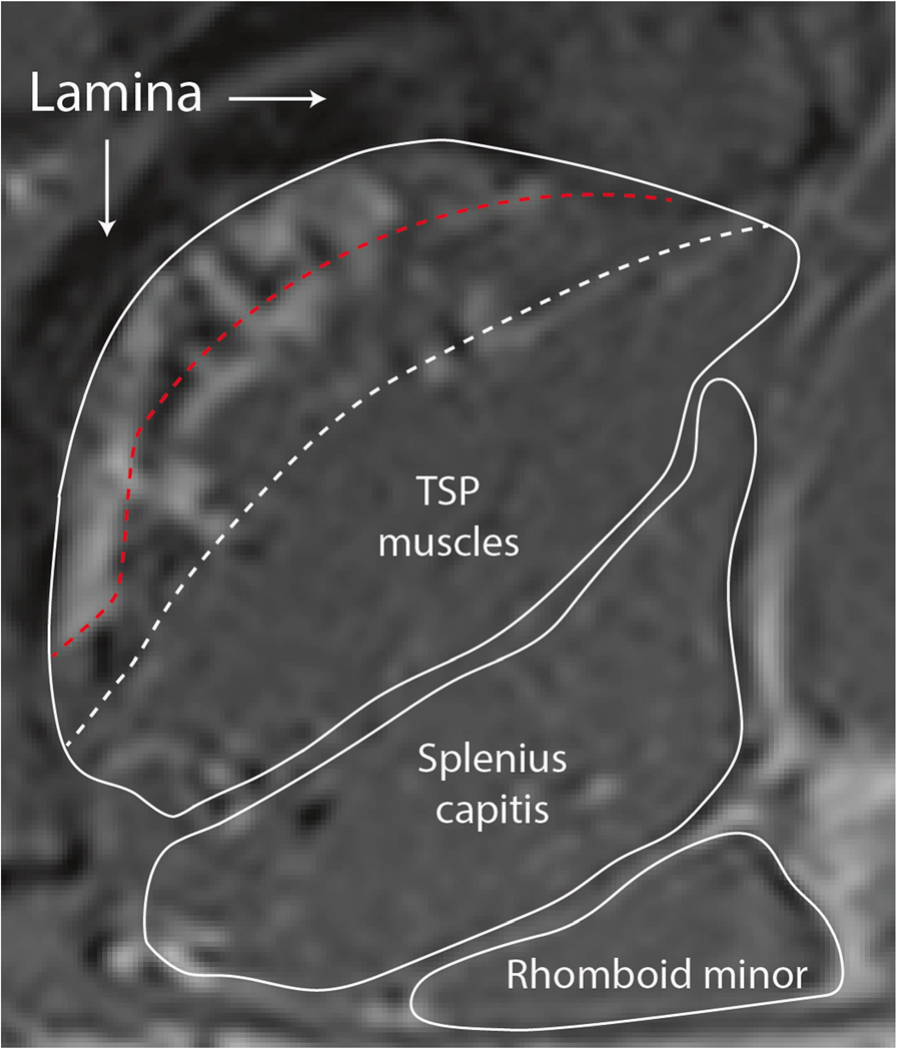
Enlarged region of posterior cervical muscles from Fig. 3 (**c**), highlighting deep boundary of region of interest (white solid line along lamina). Common mistakes for determining this region of interest for the transversospinal (TSP) muscles include the boundary of multifidus and semispinalis cervicis (white dashed line) or through the fatty infiltrate in multifidus (red dashed line)

This definition is best applied at the caudal portion of the C4 vertebral body through the entire T1 vertebral body. With current technology it is generally not possible to consistently delineate between the cervical portions of the semispinalis cervicis and multifidus on the axial slices. While measuring the two muscles independently is recommended, they can be combined to form one measure (Figs. 2, 3). As evidenced from the lumbar spine [42], the same approach of approximating the spinous process or lamina is recommended and should be included within the ROI defining cervical multifidus (Fig. 4). A challenge for both novice and expert clinicians remains what to do when creating ROIs between the spinous processes. Whether referencing the lumbar [42] or cervical spine, fat commonly overlies the interspinous space, remains defined, and should be included when generating ROIs on these slices. Finally, when the interspinous ligaments are clearly distinct with a slightly irregular and darkened edge, their lateral contour can be followed rather than the spinous process in defining the medial border [42].

#### Semispinalis capitis

This definition is best applied at the occiput-C6 levels. The muscle forms a distinct anatomical layer and can be traced consistent with the anatomy described. As semispinalis capitis is clearest at higher levels, difficulties identifying this muscle at lower cervical levels would benefit from reviewing and toggling between multiple slices from superior to inferior. As the E12 slices highlight, it may not be realistic to identify this muscle below approximately C7.

#### Longissimus capitis

This definition is best applied at the C1–4 levels. Longissimus capitis is most easily identified as a rounded muscle at its superior extent, just below the mastoid process. Towards C4 the muscle flattens, and below approximately C4 it becomes difficult to distinguish from other muscles.

#### Splenius capitis and cervicis

This definition is best applied at the C1-T3 levels. Splenius capitis is identifiable as a distinct layer located between trapezius / sternocleidomastoid and the semispinalis capitis. Care is needed around the level of the mastoid process not to confuse the superior extent of splenius capitis with sternocleidomastoid or longissimus capitis, which share attachment to the mastoid process. Just below the mastoid process at the level of C1/2 the muscles appear closely layered from superficial to deep: sternocleidomastoid, splenius capitis (both an elongated comma shape), and longissimus capitis (rounded in appearance). Below this level the muscles diverge. Ideally, splenius cervicis will be able to be distinguished from splenius capitis at the levels of C2–6 (Figs. 1, 2). However, this may not be realistic with current MRI technology. In this situation, it is reasonable to include splenius capitis and cervicis together as a single ROI.

#### Levator scapulae

This definition is best applied at the C2- T1 levels. While not part of the intrinsic cervical spine musculature, cross-sectional views highlight the presence and size of levator scapulae throughout the cervical spine. This muscle is largest inferiorly above where it arises from the upper part of the medial scapula border, and as such is best tracked superiorly from this point. Care is needed to distinguish levator scapulae from serratus anterior as they converge on the scapula (Fig. 3). Attention to slices above and below the level of interest will help resolve their borders.

### MR imaging - operational parameters

The type, quality, and output of images acquired from MR scans are highly influenced by many factors including, but not limited to, user-prescribed parameters. Similar to our previous paper covering the lumbar spine, [42] we endorse consistency in the adoption of MR imaging parameters to facilitate standardised operational procedures that allow intra-study/−institutional comparison and future pooling of results for meta-analyses.

The parameters listed here are based on those widely utilised in literature (refer to Table 1), and are adapted from those published in a previous paper on ROI for lumbar spine muscles [42]. The parenthetical values given with each parameter are not definitive or unique to a cervical spine study; rather they are displayed as an example of the consistent reporting style we propose. At a minimum, we believe the following information should be reported in all submitted manuscripts: Field strength (e.g. 3 Tesla); sequence type (e.g. 2-point DIXON (3D fast-field echo T1) whole body); repetition time (e.g. TR 4.2 ms); echo time (e.g. TE 1.2 and 3.1 ms); flip angle (e.g. 5°); field of view (e.g. FOV 560 × 352 mm); acquired voxel dimensions (e.g. 2.0 × 2.0 × 4.0 mm); reconstructed voxel dimensions (e.g. 1.0 × 1.0 × 2.0 mm); bandwidth (e.g. 240 Hz/Px), acquisition time (e.g. TA 5 min 22 s) and slice thickness (e.g. 4.0 mm). Additionally, the description should include axial slice alignment (e.g. *aligned parallel to C2–3 intervertebral disc*), slice selection (e.g. *measurements taken at most cephalad slice per vertebral level*), and subject body position including any support materials that may influence cervical spine posture/curvature (e.g. *subjects positioned supine with arms at sides and 2 in. foam cushion under head*).

## Discussion

A foundational edict for defining lumbar paravertebral ROI’s from MRI studies has previously been published [42]. Here, we expand the previous methods [57] for the cervical pre- and para-vertebral muscles using a number of MRI and E12 sheet plastinate illustrations of vertebral morphology with the aim of standardising muscle ROI definitions. The E12 plastinates provide a unique opportunity to detail specific tissues that may be MR invisible, [65] leading to natural disagreement across studies where fat-water separation is a target. Also unique to this work is the included suggestions on operational characteristics for acquiring MR images.

Similar to the proposed approach in the lumbar spine, [42] we consider that if fat is occupying space deep to the epimysial sheath and close to the spinous processes, laminae, zygapophyseal joints, it has a potential biomechanical consequence on muscle function, [6, 67] and should be included in the ROI (Fig. 4). We base this decision in part on previous work in the cervical spine [3, 5, 6, 27, 41]. Such an approach has revealed not only improved inter- and intra-rater reliability when following the spinous process and/or lamina in the cervical spine, but also the ability to discriminate between clinical groups [6]. This improved repeatability for defining MF over ES in the lumbar spine has also been demonstrated [38].

### Measures of muscle size and fat

Measures of muscle size are frequently reported in MRI and other imaging-based studies (e.g. ultrasound). In both the lumbar and cervical regions, methods employing a single cross-sectional MR slice are time efficient for determining muscle size and fat proportion within an ROI. However, a CSA measure from a single-slice should not be taken to constitute a whole muscle size or fat measure [15, 68]. Accordingly, volumetric measures, may be more appropriate [15, 69, 70]. We therefore recommend a multi-slice approach that derives muscle size and fat content based on a three-dimensional volume across the levels of interest. In going forward, such measures should be accurately categorised as a 3-dimensional volume of the entire muscle as 3D acquisition methods with MRI have evolved and are not as sensitive to the radio frequency slice profile as is 2D imaging [15].

It is of course acknowledged that acquiring such data with both semi-automated or automated programmes for both the lumbar [42] and cervical spines is time-consuming. However, with the evolution of higher-resolution imaging techniques a more time-efficient capture of cervical muscle volumes from a single vertebral level may correspond to a representative marker of MFI across the entire cervical column. While this has been demonstrated in the healthy lumbar spine [29] where the fat content at L4 best represents that of the entire lumbar region, future research should continue to systematically include the entire cervical spine in healthy and symptomatic cohorts to build a stronger body of evidence regarding age-aggregated cervical paravertebral muscle composition.

Another issue with longitudinal designs, where muscle measures are produced over time, remains a general lack of reporting on how the MRI slices are aligned in plane. A failure to do so could potentially result in registration discrepancies depending how the angle through each muscle was performed. Using some standard anatomical reference (e.g. vertebral bone) that is not expected to appreciably change over time could control for this. Errors of this type can be further minimised by reporting muscle volume over the full length of the muscle (from origin to insertion), as suggested above, rather than a single-slice CSA.

### Measures of muscle fat with MRI

The demonstration of neck muscle fatty infiltrates on T1- weighted imaging in acute [2, 3] and chronic traumatic neck pain [1, 5, 8, 30] has been reported in cross-sectional and longitudinal fashion and across three countries (Australia, [2] Sweden, [5] and the United States [3, 6]). Such findings are not present to the same magnitude for those with chronic idiopathic neck pain [7] and it has been postulated that these muscle changes represent one neurophysiologic basis for the transition to chronic pain in this population [71]. A variety of newer and more rapid high resolution MRI techniques (3D Fat/Water Separation and Proton-Density Fat Fraction, Fat suppression) [65, 72–77] and analyses (FCSA/CSA, Fat Signal Fraction, MFI %) could help better visualise and quantify physiologic changes at the level of the muscle cell or other disease processes when compared to other conventional clinical imaging sequences (e.g. T1- and T2-weighted). However, such variety across methods and techniques also complicates comparison among studies. Accordingly, we call for all authors to clearly detail their fat infiltration measurements to ensure that future pooling of data efforts is possible. Further, with the number of proprietary semi-automated or automated methods appearing in the literature, and of which descriptions are limited due to commercial sensitivity, we contend it will be helpful for authors to include enough technical detail for comparisons to the fundamental literature to be made.

### Participant positioning

It is our recommendation that participants should lie supine inside the magnet with a foam pad under their knees and foam padding placed on the right and left of the head to minimise head movement. A neutral position, visually determined by ensuring that a horizontal position of the forehead to the chin is parallel to the MRI table, is also recommended.

## Conclusion

We follow on from, and have expanded, an original paper of manually defining ROIs of lumbar spine musculature [42] to now include the cervical muscles. While the method aims to permit accurate and reliable comparison of cervical muscle quality between studies in (and beyond) this field, we further suggest journals adopt a more robust reporting of imaging parameters used to assist consistency and allow accurate comparison between studies.

It is imperative to note that we are cognisant the application methods are not definitive end-points on ‘how to’ and that there is potential for much repetition across body regions. Rather, we hope that with time, and new research findings, these methods will be modified, expanded, and refined and ultimately result in an established common methodology towards facilitating consistent and accurate definitions of lumbar, cervical, and upper/lower limb muscle ROIs on axial imaging, particularly MRI.

## Abbreviations

CSA: Cross-sectional area
FCSA: Functional cross-sectional area
FSPGR: Fast-spoiled gradient echo
IC: Iliocostalis cervicis
IP: In-phase (water)
IS: Interspinalis cervicis
IVD: Intervertebral disc
LCap: Longus capitis
LCol: Longus colli
LoCap: Longissimus capitis
LoCerv: Longissimus cervicis
LS: Levator scapulae
MF: Multifidus
MFI: Muscle fat infiltration
OP: Opposed-phase (Fat)
SCap: Spinalis capitis
SCerv: Spinalis cervicis
SCM: Sternocleidomastoid
SI: Signal intensity
SpCap: Splenius capitis
SSCap: Semispinalis capitis
SSCerv: Semispinalis cervicis
UT: Upper trapezius
